# Pendular energy transduction within the step during human walking on slopes at different speeds

**DOI:** 10.1371/journal.pone.0186963

**Published:** 2017-10-26

**Authors:** Arthur H. Dewolf, Yuri P. Ivanenko, Francesco Lacquaniti, Patrick A. Willems

**Affiliations:** 1 Laboratory of Biomechanics and Physiology of Locomotion, Institute of NeuroScience, Université Catholique de Louvain, Louvain-la-Neuve, Belgium; 2 Laboratory of Neuromotor Physiology, IRCCS Santa Lucia Foundation, Rome, Italy; 3 Department of Systems Medicine and Center of Space BioMedicine, University of Rome Tor Vergata, Rome, Italy; Northwestern University, UNITED STATES

## Abstract

When ascending (descending) a slope, positive (negative) work must be performed to overcome changes in gravitational potential energy at the center of body mass (COM). This modifies the pendulum-like behavior of walking. The aim of this study is to analyze how energy exchange and mechanical work done vary within a step across slopes and speeds. Ten subjects walked on an instrumented treadmill at different slopes (from -9° to 9°), and speeds (between 0.56 and 2.22 m s^-1^). From the ground reaction forces, we evaluated energy of the COM, recovery (i.e. the potential-kinetic energy transduction) and pendular energy savings (i.e. the theoretical reduction in work due to this recovered energy) throughout the step. When walking uphill as compared to level, pendular energy savings increase during the first part of stance (when the COM is lifted) and decreases during the second part. Conversely in downhill walking, pendular energy savings decrease during the first part of stance and increase during the second part (when the COM is lowered). In uphill and downhill walking, the main phase of external work occurs around double support. Uphill, the positive work phase is extended during the beginning of single support to raise the body. Downhill, the negative work phase starts before double support, slowing the downward velocity of the body. Changes of the pendulum-like behavior as a function of slope can be illustrated by tilting the 'classical compass model' backwards (uphill) or forwards (downhill).

## Introduction

In human walking, kinetic (*E*_k_) and gravitational potential (*E*_p_) energies of the center of mass of the body (COM) are largely out of phase, resulting in an exchange between these two forms of energies [1]. The present study is intended to provide a detailed description of the change in the fluctuation of the energy of the COM, by assessing the changes in pendulum-like exchange between *E*_k_ and *E*_p_, and in the mechanical work required to sustain the motion of the COM (*W*_ext_) with slope of the terrain and speed of progression during human walking.

The exchange between *E*_k_ and *E*_p_ appears after the age of one year [2, 3]. In adults, the fluctuation of the energy of the COM is known to change in various walking conditions: with walking speed [1], with the softness of the ground [4] or with the slope of the terrain [5–7]. Furthermore, African women are able to carry head-supported loads of up to 20% of their body weight for ‘free’ while enhancing the *E*_k_-*E*_p_ transduction [8, 9].

The *E*_k_-*E*_p_ transduction is classically evaluated over an entire step by measuring the recovery, *R*_step_ [1], defined as the ratio between the work theoretically saved by the pendulum-like energy exchange and the work theoretically performed assuming no energy transduction. When walking on the level ground, *R*_step_ varies with speed, in part because the amplitudes of the *E*_k_-time and of the *E*_p_-time curves change differently with speed [1].

When walking on a slope at a steady speed, the trajectory of the COM is modified since the body must gain/lose height each stride. This change in the trajectory will affect the pendulum-like exchange between *E*_p_ and *E*_k_, since the average level of *E*_p_ changes continuously whereas the average level of *E*_k_ remains constant. Consequently, *R*_step_ is also altered when walking on a slope.

Minetti et al. [10], Minetti and Ardigò [11] and Gottschall and Kram [5] observed that the *E*_k_-*E*_p_ transduction still occurs during walking even on steep positive or negative slopes. As compared to level walking, they reported that the *E*_k_-*E*_p_ exchange is reduced when ascending. When descending, Gottschall and Kram [5] observed an increase of the energy transduction. Note that these authors estimated the pendular energy transduction after factoring out the changes in *E*_p_ due to slope, which cancels the asymmetric fluctuation of the *E*_p_ *vs* time curve. In two recent studies, Gomeñuka et al. [6] and Gomeñuka et al. [7] have also observed that *R*_step_ is reduced and that the speed at which *R*_step_ is maximal is higher when walking uphill. However, it remains unclear why *R*_step_ is reduced and why the speed at which *R*_step_ is maximal is increased.

The variable *R*_step_ is computed over the whole step and does not help understanding how the *E*_k_-*E*_p_ transduction changes throughout the duration of a step. To our knowledge, no study has measured the time course of this transduction. For this purpose, we have calculated the ‘within-step recovery', *r*(*t*), which evaluates the *E*_p_-*E*_k_ transduction at each instant *t* of the walking step [8]. In this way, it is possible to separately assess the transduction of *E*_p_ into *E*_k_ and of *E*_k_ into *E*_p_ during the different phases of the step.

Perfect energy recovery of 100% at any phase of the step cycle would indicate that the changes in potential energy mirror the changes in kinetic energy. However, this does not provide any insight into the magnitude of mechanical energy changes (in Joules). Therefore, we introduce here a new variable: the theoretical 'pendular energy savings' within the step (*E*_s_), which represents the magnitude of energy exchanged, i.e. the difference between the sum of *E*_p_ and *E*_k_ assuming no transduction and the external work actually done when the *E*_p_-*E*_k_ transduction occurs. In other words, the new parameter *E*_s_, designed to quantify the effectiveness of the transduction between *E*_p_ and *E*_k_ in the different phases of the step, extends the information given by *r*(*t*). Indeed, *E*_s_ offers the possibility to determine the periods of the step where, despite high *r*(*t*), the *E*_p_- and *E*_k_-curves are flattened, resulting in a small amount of energy exchange.

Despite the pendulum-like exchange of energy, work must be done to sustain the movement of the COM relative to the surroundings. For example, when analyzing the energy curves of the COM, positive work must be done during two phases of the step: the first (named phase *a* by Cavagna et al [1]) occurs close to the bottom of the COM trajectory to give a push forward, and the second one (named phase *b*) occurs close to the top of the COM trajectory to complete its vertical lift. According to Kuo et al. [12] mechanical work is predominantly required to redirect the COM velocity vector during step-to-step transitions. Furthermore, the continuous changes in potential energy on slopes affect the amount and the time pattern of positive or negative work production. Therefore, the amount of positive and negative external work performed throughout the step was also measured.

The present study is intended to provide more quantitative details on the time-varying changes of the energy at COM during each step, when walking on various slopes at different speeds. This is done by including an estimate of the exchange of energy and of the amount of energy saved, at each instant of the walking step. Therefore, the following variables are assessed: (1) the 'within-step recovery', which quantify the out-of-phase fluctuation of *E*_p_ and *E*_k_, (2) the ‘pendular energy savings', which evaluates the effectiveness of the transduction that occurs and (3) the positive and negative external work done despite energy savings. Identification of the changes of *E*_p_-*E*_k_ transduction and external work production shed light on one potential mechanism that could minimize muscle work during slope ascent and descent.

## Methods

### Subject and experimental procedure

Ten healthy subjects (6 ♂ & 4 ♀, age: 22.2 ± 2.4 y, mass: 69.2 ± 14.4 kg, height: 1.75 ± 0.10 m, mean ± SD) gave their written informed consent to participate to the experiences. The study followed the guidelines of the Declaration of Helsinki, and the procedures were approved by the Ethic Committee of the Université catholique de Louvain (Ref. number: 2015/06JUL/372).

Subjects walked on an instrumented treadmill mounted on wedges of different inclinations: 0°, 3°, 6° and 9° (corresponding to 0%, 5.2%, 10.5%, 15.8% respectively). Note that some subject did not walk at all slopes (Table 1). Each trial lasted about 45 s and data were recorded during the last 30 s. At each slope, half the subjects started with uphill walking and the other half with downhill walking. In order to neutralize the effect of learning and muscle fatigue, half of the subjects started with an inclination of 0° that was progressively increased and the other half with an inclination of 9° that was progressively decreased. Furthermore, the resting period between each trial lasted at least 3’ (>15’ when the slope of the treadmill had to be changed). At each slope, subjects walked at 7 speeds (from 0.56 to 2.22 m s^-1^, i.e. 2 to 8 km h^-1^). Some subjects started to run at the highest speed and these trials were rejected (Table 1). Between 6 and 36 strides per trials were recorded (Table 2) and a total of 8,864 strides were analyzed.

**Table 1:** Number of subjects in each slope/speed class.

	-9°	-6°	-3°	0°	3°	6°	9°
**0.56 m s**^**-1**^	8	8	6	10	6	8	8
**0.83 m s**^**-1**^	8	8	6	10	6	8	8
**1.11 m s**^**-1**^	8	8	6	10	6	8	8
**1.39 m s**^**-1**^	8	8	6	10	6	8	8
**1.67 m s**^**-1**^	8	8	6	10	6	8	8
**1.94 m s**^**-1**^	8	8	6	10	6	8	8
**2.22 m s**^**-1**^	5	6	5	8	5	6	5

**Table 2:** Minimum and maximum number of steps averaged for each subject in each slope/speed class.

	-9°	-6°	-3°	0°	3°	6°	9°
**0.56 m s**^**-1**^	22/36	20/40	12/24	12/36	22/32	26/34	14/36	
**0.83 m s**^**-1**^	38/48	40/46	36/44	26/44	36/42	32/44	36/44	
**1.11 m s**^**-1**^	42/56	46/52	46/54	42/50	44/48	42/48	42/50	
**1.39 m s**^**-1**^	42/62	50/56	48/58	48/54	48/54	44/54	46/54	
**1.67 m s**^**-1**^	34/60	52/60	52/64	44/60	50/58	50/58	50/60	
**1.94 m s**^**-1**^	58/66	54/66	56/68	44/66	54/64	48/62	56/66	
**2.22 m s**^**-1**^	50/68	48/68	42/62	60/72	60/70	60/68	52/66	

### Experimental setup and data analysis

The instrumented treadmill consisted of a modified commercial treadmill (h/p/Comos-Stellar, Germany). The whole treadmill was mounted on four strain-gauge force transducers (Arsalis, Belgium) that measure the three components of the force exerted by the feet on the belt (GRF): *F*_p_, the component parallel to the long axis of the tread-surface, *F*_n_ the component normal to the tread-surface and *F*_l_ the component in the lateral direction [13]. Data were sampled at a frequency of 500 Hz. From these data, the vertical (*F*_v_) and horizontal forward (*F*_f_) component of the GRF were computed as:

$$\left(\begin{array}{c} F_{f}\\ F_{v} \end{array}\right)=\left(\begin{array}{cc} \cos\theta & \text{-sin}\theta \\ \sin\theta & \cos\theta \end{array}\right)\;\left(\begin{array}{c} F_{p}\\ F_{n} \end{array}\right),$$ (1)

where *θ* is the angle of the treadmill relative to level ground.

The acceleration, velocity and the displacement of the COM were determined from the GRF using the procedure described in detail in Dewolf et al. [13]. In short, the horizontal, lateral and vertical acceleration of the COM were respectively computed as *a*_f_ = *F*_f_ /*m*, *a*_l_ = *F*_l_ /*m* and *a*_v_ = (*F*_v_-*m g*)/*m*, where *m* is the subject’s body mass and *g* is acceleration due to gravity. The instantaneous velocity in the forward (*v*_f_), lateral (*v*_l_) and vertical (*v*_v_) directions were obtained by integration of *a*_f_, *a*_l_ and *a*_v_ plus an integration constant. In the fore-aft direction, this constant was chosen to make the average velocity over all the strides of the trial equal to *v*_belt_ cos*θ* (*v*_belt_ is the velocity of the tread-belt measured with an optical encoder). In the two other directions, constants were chosen to make the average lateral velocity over each stride equal to zero and the average vertical velocity over each stride equal to *v*_belt_ sin*θ*. A second integration of the vertical velocity yields the vertical displacement of the COM.

#### Division of the step

Steps were divided according to the maximum of *v*_f_. Steps were grouped in strides composed of a right step followed by a left step. A stride was considered to be suitable for analysis if (1) ${\bar{F}}_{v}$ was within 5% of body weight and (2) the sum of the increments and the sum of the decrements of *v*_f_ differed less than 25%. Foot contact and toe-off were estimated from the displacement of the center of pressure on the belt [14].

#### Estimates of the positive and negative external work

The energy of the COM (*E*_ext_) was calculated from the GRF following the methods of Gosseye et al. [15]; *E*_ext_ was the algebraic sum at each instant of its gravitational potential energy *E*_p_ and of its kinetic energy *E*_k_. *E*_k_ was calculated as the sum of *E*_kf_, the kinetic energy due to the forward movements of the COM, *E*_kl_ the kinetic energy due to its lateral movements and *E*_kv_ the kinetic energy due to its vertical movements.

**Fig 1:**
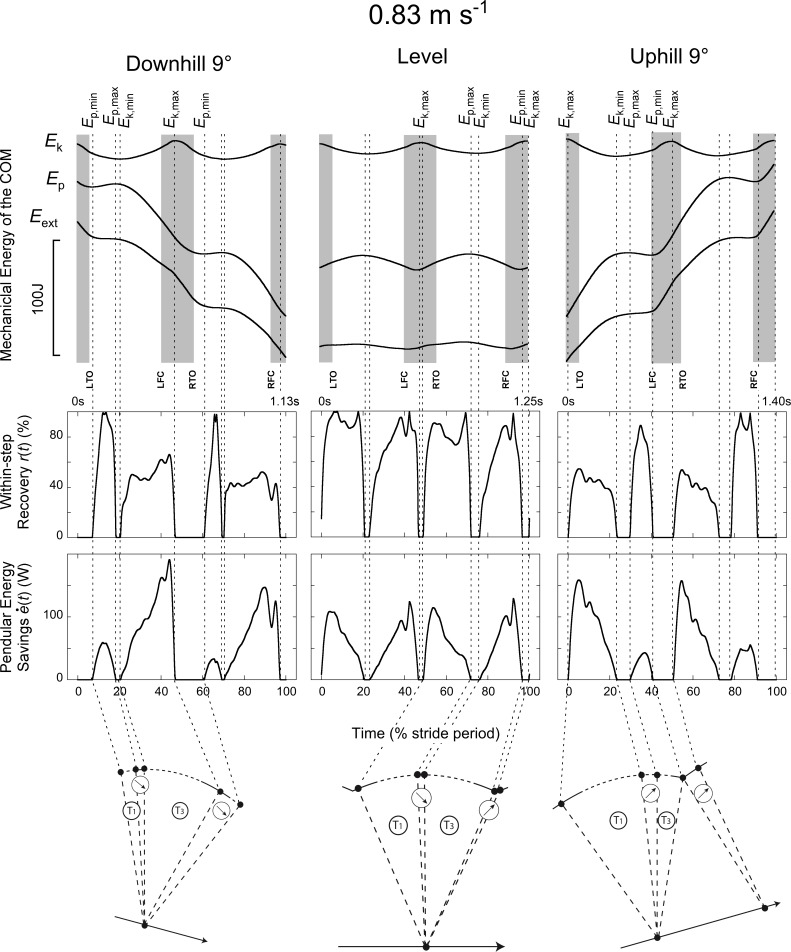
Typical time-traces of a subject walking at ~0.83 m s^-1^ (3 km h^-1^) on a -9° slope (left column), on the level (middle column) and on a +9° slope (right column). **Top panels:** Mechanical energy-time curves of the COM during a stride. Strides are delimited by the maximal fore-aft velocity of the COM when the right foot is in front. The upper curve (*E*_k_) refers to the kinetic energy of the COM, the middle curve (*E*_p_) to its gravitational potential energy and the bottom curve (*E*_ext_ = *E*_k_ + *E*_p_) to the total energy of the COM. The grey zones correspond to the double contact periods (starting at right or left foot contact -RFC or LFC- and ending at right or left toe-off -RTO or LTO). The vertical interrupted lines correspond to the extrema of the *E*_k_ and *E*_p_ curves. Note that these energy-time curves are qualitatively similar to the one obtained by Gottschall and Kram (2006). **Middle panels:** Instantaneous recovery (*r*(*t*), Eq 3) at each instant *t* of the stride. The recovery *r*(*t*) varies form 0% when the *E*_k_ and *E*_p_ curves are in phase, to 100% when the decrease of one curve equals the increase of the other. When *r*(*t*) = 100%, *E*_ext_ is constant and no external work is done to move the COM relative to the surroundings. **Lower panels:** Rate of pendular energy savings ($\dot{e}(t)$, Eq 4) at each instant *t* of the stride. These curves represent the rate at which energy is economized through the transformation of kinetic into potential energy (or *vice versa*). Time is expressed as a percentage of the stride period. Tracings were recorded on a male subject (height: 1.82 m, body mass: 74.3 kg, age: 24.7 y.o.). **Lower schemas:** The schemas represent the 'compass walking' model with the approximate division of one step of the stride into the four phases T1, T2, T3 & T4 (see methods) during downhill, level and uphill walking. During T1, *E*_k_ decreases while *E*_p_ increases, during T3 *E*_k_ increases while *E*_p_ decreases. During T2 and T4, both *E*_k_ and *E*_p_ increase or decrease (arrows).

**Fig 2:**
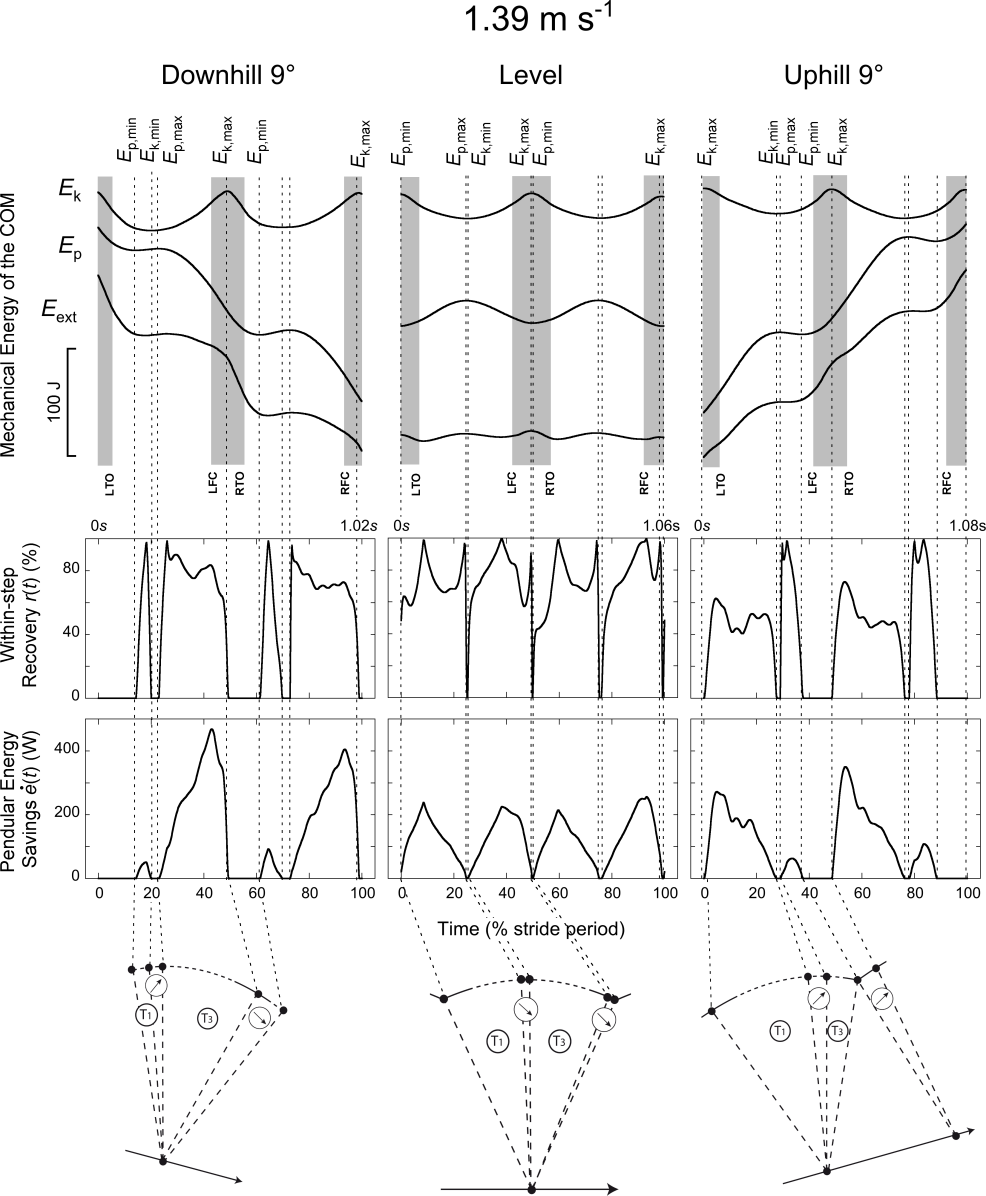
Typical time-traces of a subject walking at ~1.39 m s^-1^ (5 km h^-1^) on a -9° slope (left column), on the level (middle column) and on a +9° slope (right column). Same indications as in Fig 1.

**Fig 3:**
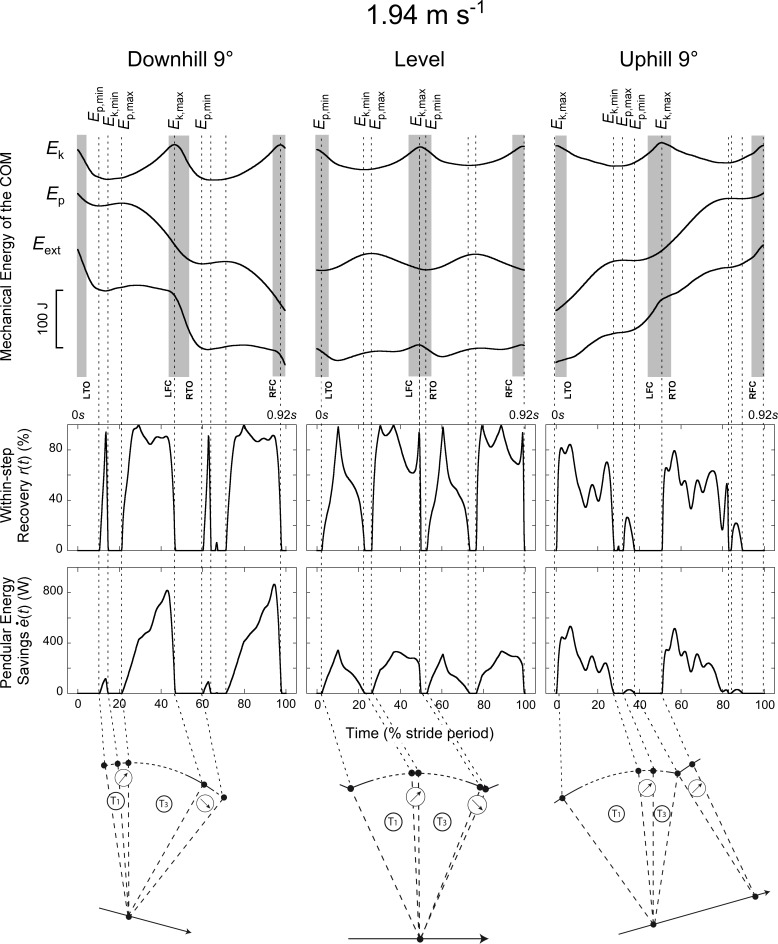
Typical time-traces of a subject walking at ~1.94 m s^-1^ (7 km h^-1^) on a -9° slope (left column), on the level (middle column) and on a +9° slope (right column). Same indications as in Fig 1.

External work is defined here as the work necessary to move the COM relative to the surroundings [16], i.e. the work needed to increase and decrease energy of the COM. Positive ($W_{ext}^{+}$) and negative ($W_{ext}^{-}$) COM work was computed by summing the increments and the absolute value of the decrements of the *E*_ext_ curve, respectively. Similarly, $W_{k}^{+}$, $W_{k}^{-},\;W_{p}^{+}$ and $W_{p}^{-}$ were computed by summing respectively the increments and the absolute value of the decrements of the *E*_k_ and *E*_p_ curves (Figs 1, 2 & 3). Increments in the curves were considered to represent work only if the time between two successive extrema was greater than 20 ms. Based on the data of this study, the work done on/by the belt of the treadmill is <2% of ($W_{ext}^{+}+|W_{ext}^{-}|$). Therefore, it was not taken into account.

#### Recovery over the step

To evaluate the pendulum-like energy exchange, Cavagna et al. [1] proposed to estimate the relative amount of energy recovered by the transduction between energy due to the vertical motion of the COM (*E*_p_+*E*_kv_) and energy due to its forward motion (*E*_kf_). Here, we have also added the work due to the lateral movement of the COM. The relative amount of energy saved over a step (*R*_step_ in %) can then be computed as:

$$R_{\mathrm{step}}=100\frac{W_{f}^{+}+|W_{f}^{\text{-}}|+W_{v}^{+}+|W_{v}^{\text{-}}|+W_{l}^{+}+|W_{l}^{\text{-}}|-[W_{\mathrm{ext}}^{+}+|W_{\mathrm{ext}}^{\text{-}}|]}{W_{f}^{+}+|W_{f}^{\text{-}}|+W_{v}^{+}+|W_{v}^{\text{-}}|+W_{l}^{+}+|W_{l}^{\text{-}}|},$$ (2)

where $W_{f}^{+}$ and $W_{f}^{-}$ are the sum of respectively the increments and the absolute value of the decrements of the *E*_kf_ vs. time curve, $W_{v}^{+}$ and $W_{v}^{-}$ the sum of respectively the increments and the absolute value of the decrements of the *E*_p_+*E*_kv_ vs. time curve and $W_{l}^{-}$ and $W_{l}^{+}$ the sum of respectively the increments and the absolute value of the decrements of the *E*_kl_ vs. time curve. Of note, *R*_step_ has also been defined in prior literature based on *E*_k_ and *E*_p_ [17]. These authors have shown that this formulation yields similar results to those from Eq 2.

#### Recovery and pendular energy savings within the step

In order to identify periods within each step during which a *E*_p_-*E*_k_ transduction occurs, Cavagna et al. [8] proposed to estimate the within-step recovery at each instant *t* of the step (*r*(*t*) in %) from the absolute value of the kinetic and potential energy changes:

$$r(t)=100\;\frac{\left|E_{k}(t)-E_{k}(t-\Delta t)|+|E_{p}(t)-E_{p}(t-\Delta t)|-|E_{ext}(t)-E_{ext}(t-\Delta t)\right|}{\left|E_{k}(t)-E_{k}(t-\Delta t)|+|E_{p}(t)-E_{p}(t-\Delta t)\right|},$$ (3)

where Δ*t* is the time between two samples.

To evaluate the theoretical amount of mechanical power saved by this recovery, we introduce a new outcome, the theoretical rate of pendular energy savings, $\dot{e}(t)$, computed as:

$$\dot{e}(t)=\frac{\left|E_{k}(t)-E_{k}(t-\Delta t)|+|E_{p}(t)-E_{p}(t-\Delta t)|-|E_{ext}(t)-E_{ext}(t-\Delta t)\right|}{\Delta t}.$$ (4)

Typical COM energy, *r*(*t*) and $\dot{e}(t)$ curves are presented in Figs 1, 2 & 3. Two additional summary metrics were computed over four periods of the steps: (1) *R*_avg_ was defined as the average of *r*(*t*) over each period and (2) *E*_s_, the amount of pendular energy savings, was defined as the time-integral of $\dot{e}(t)$ over each period. Four periods (T1-T4) were defined according to the local maxima and minima of *E*_k_ and *E*_p_: the first period T1 occurs when *E*_p_ increases while *E*_k_ decreases; a subsequent period T3 occurs when *E*_p_ decreases while *E*_k_ increases; between T1 and T3 and between T3 and T1, we define the period T2 and T4 respectively, where *E*_p_ and *E*_k_ changes are in phase.

### Statistics

Data were grouped into speed-slope classes. Each stride was divided into a right and left step. In order to obtain one value per subject in each class, both right and left steps of a subject in a given class were averaged. The mean and standard deviation of the population were then computed in each class (grand mean). The variables were analyzed across all conditions using a repeated measures ANOVA with post-hoc Bonferroni correction to assess the difference between conditions (PASW Statistics 19, SPSS inc®, IBM company, USA). The normality of the residuals was checked visually with QQ-plots and was assumed for all variables. An α-threshold of 0.05 was used throughout to assess statistical significance.

## Results

### Work and recovery

**Fig 4:**
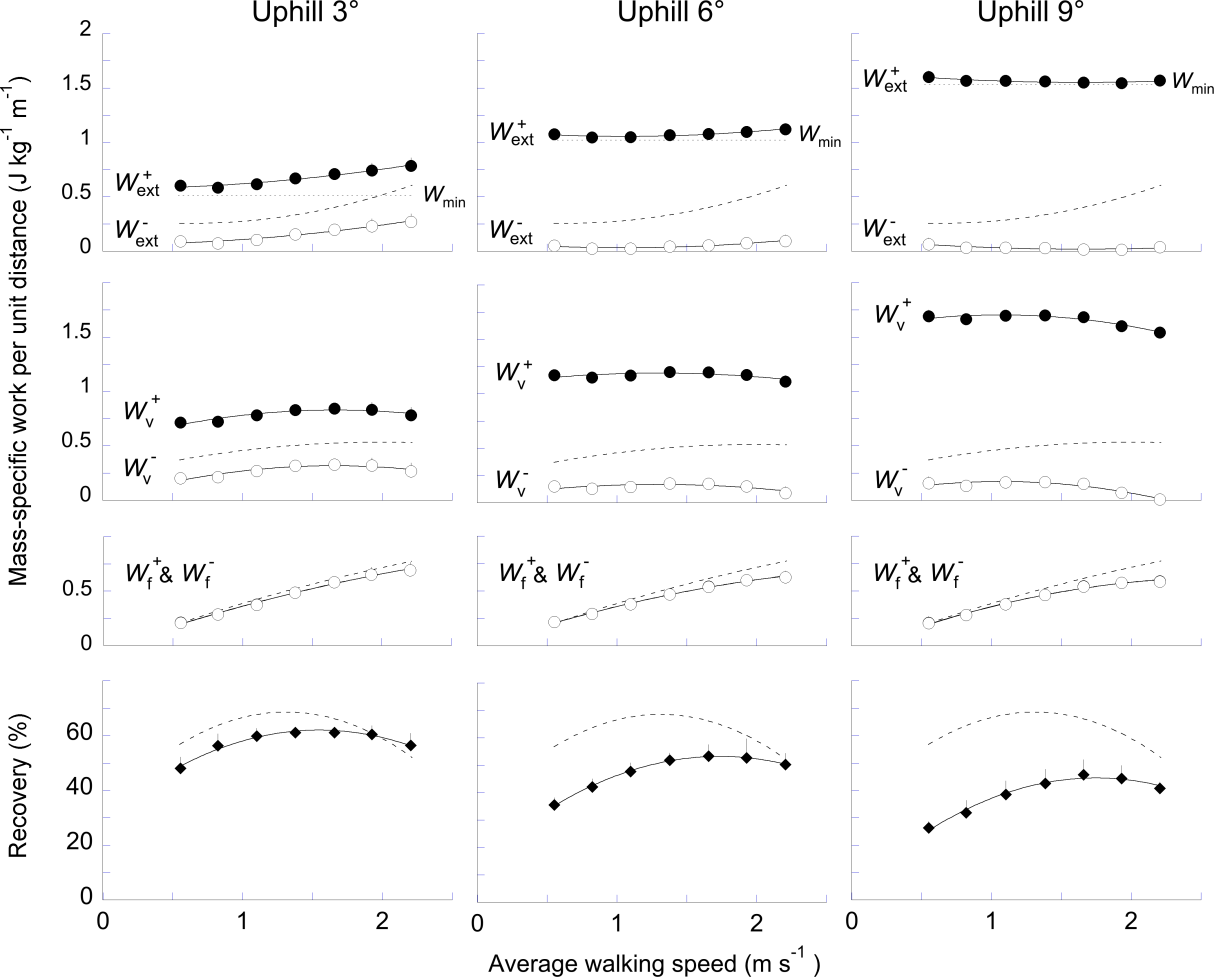
Mass-normalized work per unit distance and recovery over the step as a function of speed and slope while walking uphill. **Three top rows:** For each slope, the mass-normalized positive (closed symbols, superscript +) and negative (open symbols, superscript -) mechanical work done per unit distance is given as a function of walking speed. **Top row:** $W_{\mathrm{ext}}^{+}$ and $W_{\mathrm{ext}}^{-}$ are the positive and negative external work estimated to move the *COM* through its observed trajectory, relative to the surroundings. The horizontal dotted line (*W*_m_) represents the minimal work necessary to overcome the change in gravitational potential energy. **Second row:** $W_{v}^{+}$ and $W_{v}^{-}$ are the positive and negative work due to the changes in *E*_p_+*E*_kv_ vs. time curve. **Third row:** $W_{f}^{+}$ and $W_{f}^{-}$ are the positive and negative work due to the changes in *E*_kf_ vs. time curve (since the average speed is constant, $W_{f}^{+}=W_{f}^{-}$). Because $W_{l}^{+}$ and $W_{l}^{-}$ represents less than 1.5% of $W_{\mathrm{ext}}^{+}$ and $W_{\mathrm{ext}}^{-}$, it is not presented in this figure. **Bottom row:** The bottom panels present the recovery calculated over the whole step (*R*_step_, Eq 2) as a function of speed. In each panel, symbols and bars represent the "grand mean" of the subjects (see Methods and Table 1) and the standard deviations (when the length of the bar exceeds the size of the symbol). The continuous lines were drawn through the experimental data (polynomial function, Kaleidagraph 4.5). The dashed lines represent the work done or the recovery during walking on the level; these lines were also drawn through the experimental data (polynomial function, Kaleidagraph 4.5).

**Fig 5:**
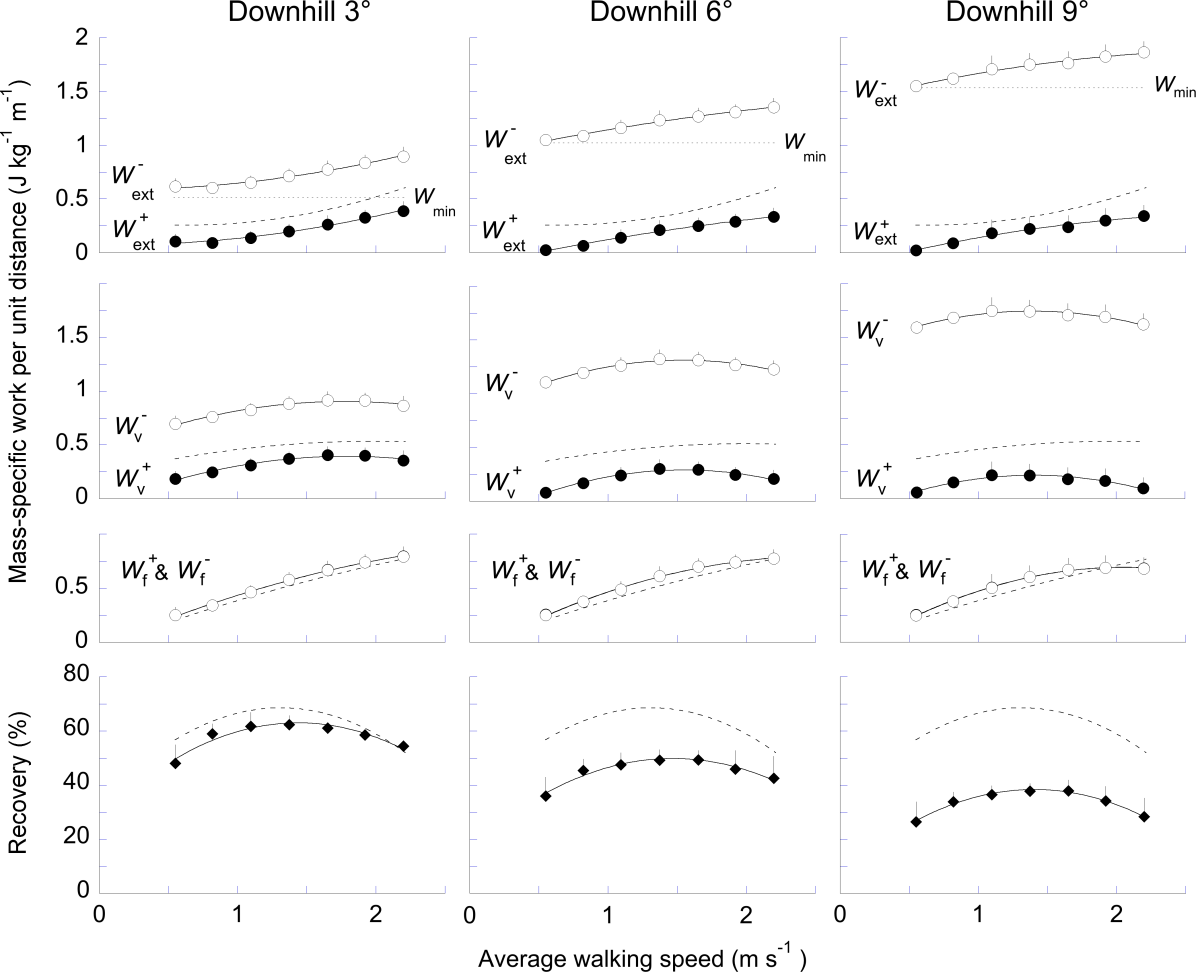
Mass-specific work per unit distance and recovery over the step as a function of speed and slope while walking downhill. Same indications as in Fig 4.

External work and recovery of mechanical energy over the whole walking step (*R*_step_) are presented as a function of speed when walking uphill (Fig 4) and downhill (Fig 5). $W_{f}^{+}$ and $W_{f}^{-}$ increase with speed at all slopes (*F*_6,363_ = 343.8, *p*< 0.001). At a given speed, since the average velocity over the successive steps is constant, $W_{f}^{+}\approx W_{f}^{-}$. However, as compared to level walking, $W_{f}^{+}$ and $W_{f}^{-}$ are each slightly larger in magnitude during downhill walking and slightly smaller during uphill walking (*F*_6,363_ = 30.2, *p*< 0.001).

When walking on a flat terrain, $W_{v}^{+}\approx W_{v}^{-}$ over each step. When walking on a positive slope, $W_{v}^{+}$ increases while $W_{v}^{-}$ decreases as compared to level walking. On a steep positive slope, $W_{v}^{-}$ is nil at the highest speed because the *E*_p_+*E*_kv_ vs. time curve increases monotonically. When walking on a negative slope, $W_{v}^{+}$ decreases while $W_{v}^{-}$ increases. On a steep negative slope, $W_{v}^{+}$ is nil at the slowest and highest speeds because the *E*_p_+*E*_kv_ vs. time curve decreases monotonically.

The difference between $W_{v}^{+}$ and $W_{v}^{-}$ on an incline causes an imbalance between $W_{\mathrm{ext}}^{+}$ and $W_{\mathrm{ext}}^{-}$. When walking uphill on a shallow slope (Fig 4), $W_{\mathrm{ext}}^{-}$ is reduced as compared to level walking. When the slope become steeper, $W_{\mathrm{com}}^{-}$ is almost nil at all speeds and the $W_{\mathrm{com}}^{+}$ is equal to the minimum work required to overcome the slope (*W*_min_). Consequently, *R*_step_ is reduced as compared to level walking: its maximum decreases from 68% on the level to 63% at +3°, to 53% at +6° and to 45% at +9°. Note that the speed at which *R*_step_ is maximal is greater than on level ground: 1.30 m s^-1^ at 0°, 1.55 m s^-1^ at +3°, 1.65 m s^-1^ at +6° and 1.75 m s^-1^ at +9°.

When walking downhill, $W_{\mathrm{ext}}^{+}$ decreases with the slope but increases slightly with speed at each gradient. $W_{\mathrm{ext}}^{+}$ is greater than *W*_min_ at all speeds (except at the lowest speed at -6° and -9°). As on a positive slope, *R*_step_ decreases when the negative slope increases: *R*_step_ decreases to 62% at -3°, to 50% at -6° and to 38% at -9°. Here also, the speed at which *R*_step_ is maximal is greater than on level ground: 1.53 m s^-1^ at -3°, 1.48 m s^-1^ at -6° and 1.46 m s^-1^ at -9°.

### Time course of the instantaneous recovery and rate of pendular energy savings within the stride

Figs 1, 2 & 3 present typical traces of *E*_k_, *E*_p_, *E*_ext_, *r*(*t*) and $\dot{e}(t)$ during a walking stride (two steps). During T1 and T3, *r*(*t*)> 0 and $\dot{e}(t)$ > 0 since *E*_k_ can be transformed into *E*_p_ or *vice-versa*; during T1, *E*_k_ decreases while *E*_p_ increases, whereas during T3, *E*_k_ increases while *E*_p_ decreases. During T2 and T4, *E*_k_ and *E*_p_ are both increasing or decreasing (depending on slope and speed of progression). Since these two curves are in phase, *r(t)* = 0 and $\dot{e}(t)$ = 0.

**Fig 6:**
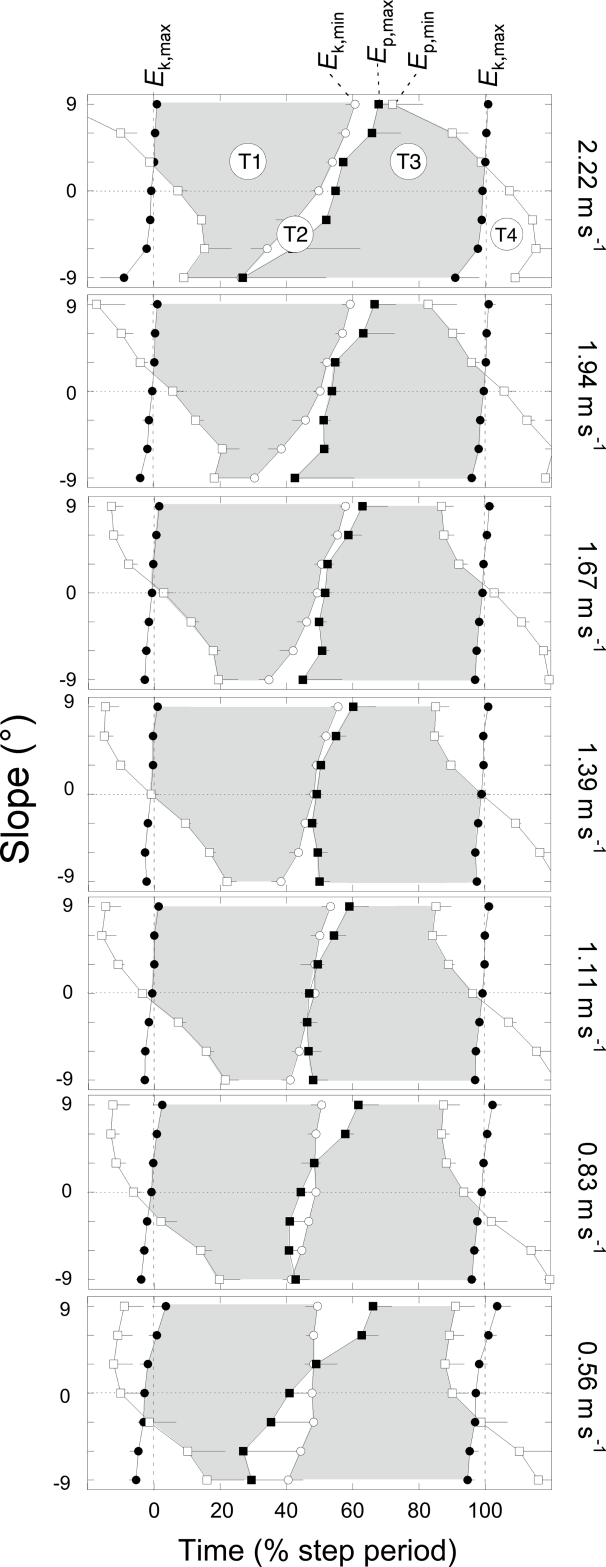
Duration of the four phases of the step as a function of slope at each speed. In each panel, the time is expressed as a percentage of the step duration: 0% and 100% (vertical interrupted lines) correspond to the moments at which the fore-aft velocity of the COM is maximal. The closed circles correspond to the instant of *E*_k,max_, the open circles to *E*_k,min_, the closed squares to *E*_p,max_, and the open squares to *E*_p,min_. The grey zones correspond to the phases during which *E*_k_ can be transformed into *E*_p_ (T1) and *vice versa* (T3). The white zones correspond to the phases where the curves are in phase. Symbols and bars represent the "grand mean" of all subjects (Table 1) at a given speed and slope and the standard deviations (when the length of the bar exceeds the size of the symbol).

When walking on a positive or negative slope, the duration of the different phases changes as compared to level walking (Figs 1, 2 & 3). When walking on a flat terrain, the duration of T1 and T3 are about equal at all speeds (Fig 6, *t* = -1.39, *p* = 0.166). On a positive slope, T3 becomes relatively shorter while T1 becomes relatively longer than at 0° slope. On a negative slope, T1 becomes shorter while T3 becomes longer. Both effects are more pronounced when walking at high speeds. At all speeds and slopes, T2 does not exceed ~10% of the step period. On the contrary, T4 increases with slope and speed: at the highest speed, T4 represents ~30% of the step on a positive slope, while it represents ~20% on a negative slope. When walking uphill both the *E*_k_ and *E*_p_ curves increase during T4 (since *E*_p,min_ precedes *E*_k,max_), while when walking downhill both curves decrease (since *E*_p,min_ follows *E*_k,max_).

### Instantaneous recovery and pendular energy savings

**Fig 7:**
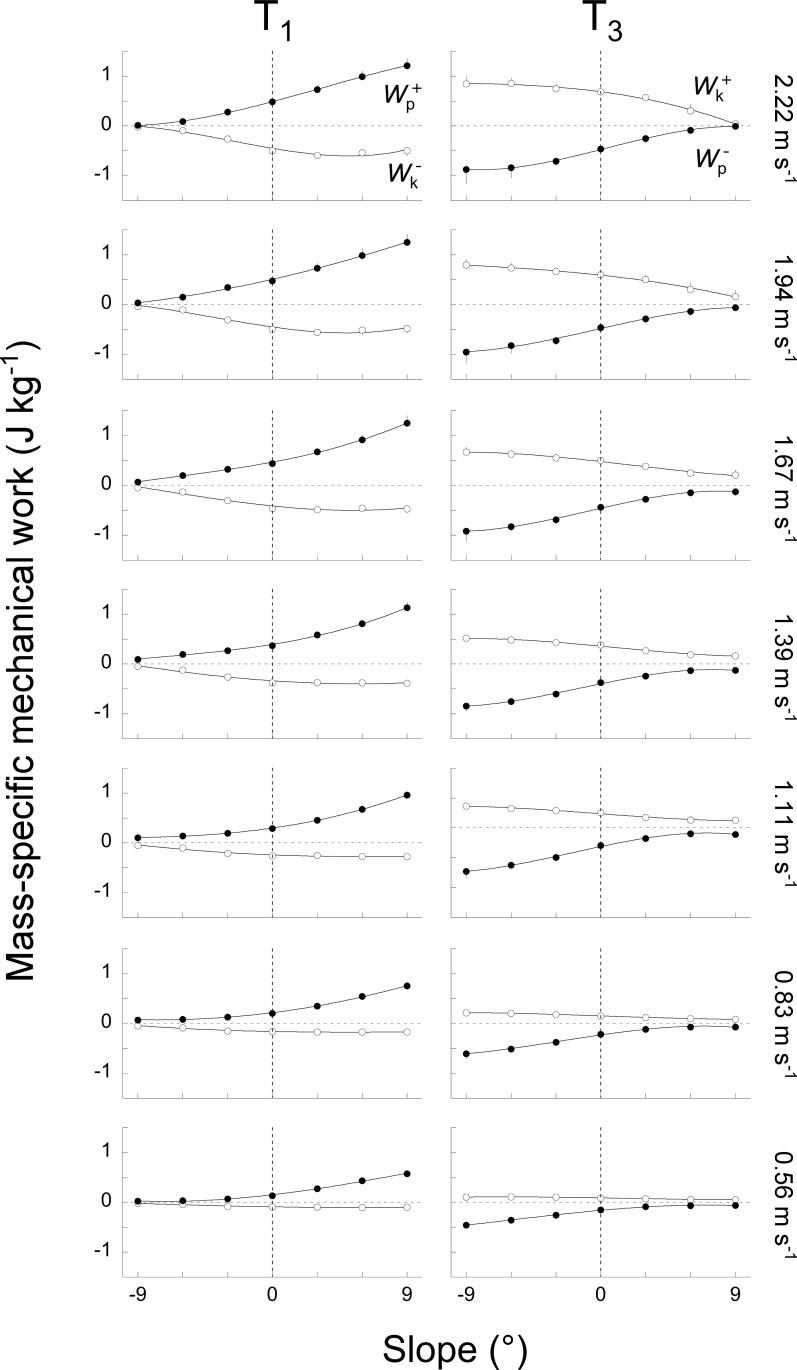
Change in the kinetic and potential energy during the periods T1 and T3 as a function of slope at each speed. At each speed, the left panel presents the increments of the *E*_p_ curve ($W_{p}^{+}$, closed circles) and the decrements of the *E*_k_ curve ($W_{k}^{-}$, open circles) during the period T1 whereas the right panel presents the decrements of the *E*_p_ curve ($W_{p}^{-}$, closed circles) and the increments of the *E*_k_ curve ($W_{k}^{+}$, open circles) over the period T3. Other indications as in Fig 4.

**Fig 8:**
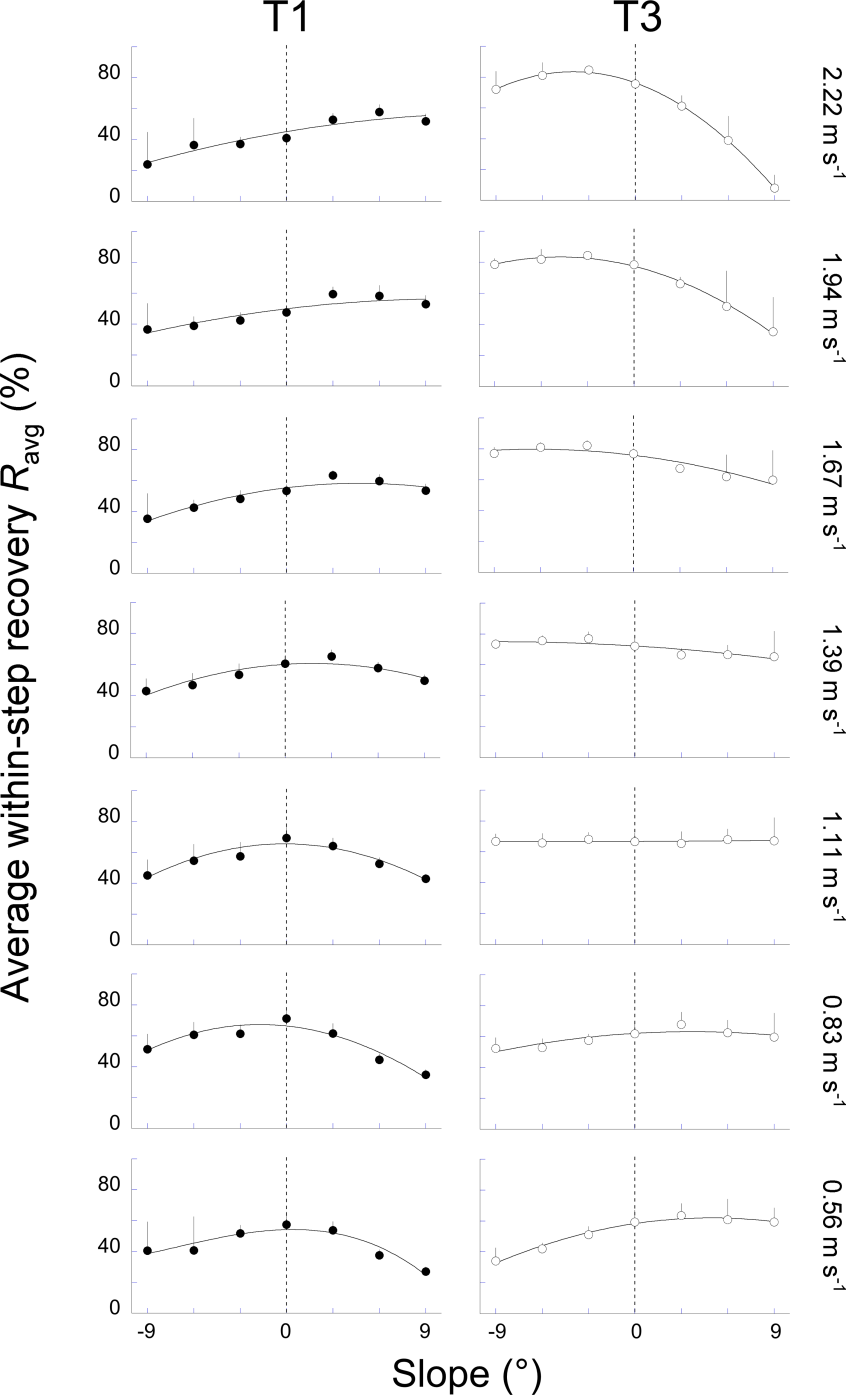
Average of the instantaneous recovery (*R*_avg_) over the periods T1 and T3 as a function of slope at each speed. At each speed, the left panel (closed circles) presents *R*_avg_ (see Methods) over the period T1 whereas the right panel (open circles) presents *R*_avg_ over the period T3. Other indications as in Fig 4.

During T1, $W_{p}^{+}$ and $W_{k}^{-}$ tend to increase from -9° to +9°, while during T3, $W_{p}^{-}$ and $W_{k}^{+}$ tend to decrease (Fig 7). These changes are more important at high speeds than at low speeds. The effect of slope seems more marked on $W_{p}^{+}$ and $W_{p}^{-}$, whereas the influence of speed is more discernable on $W_{k}^{+}$ and $W_{k}^{-}$, as one would expect. Due to the change in the magnitude of the *E*_p_ and the *E*_k_ curves, the average recovery (*R*_avg_) during each period changes with slope and speed (Fig 8).

#### Uphill walking

**Fig 9:**
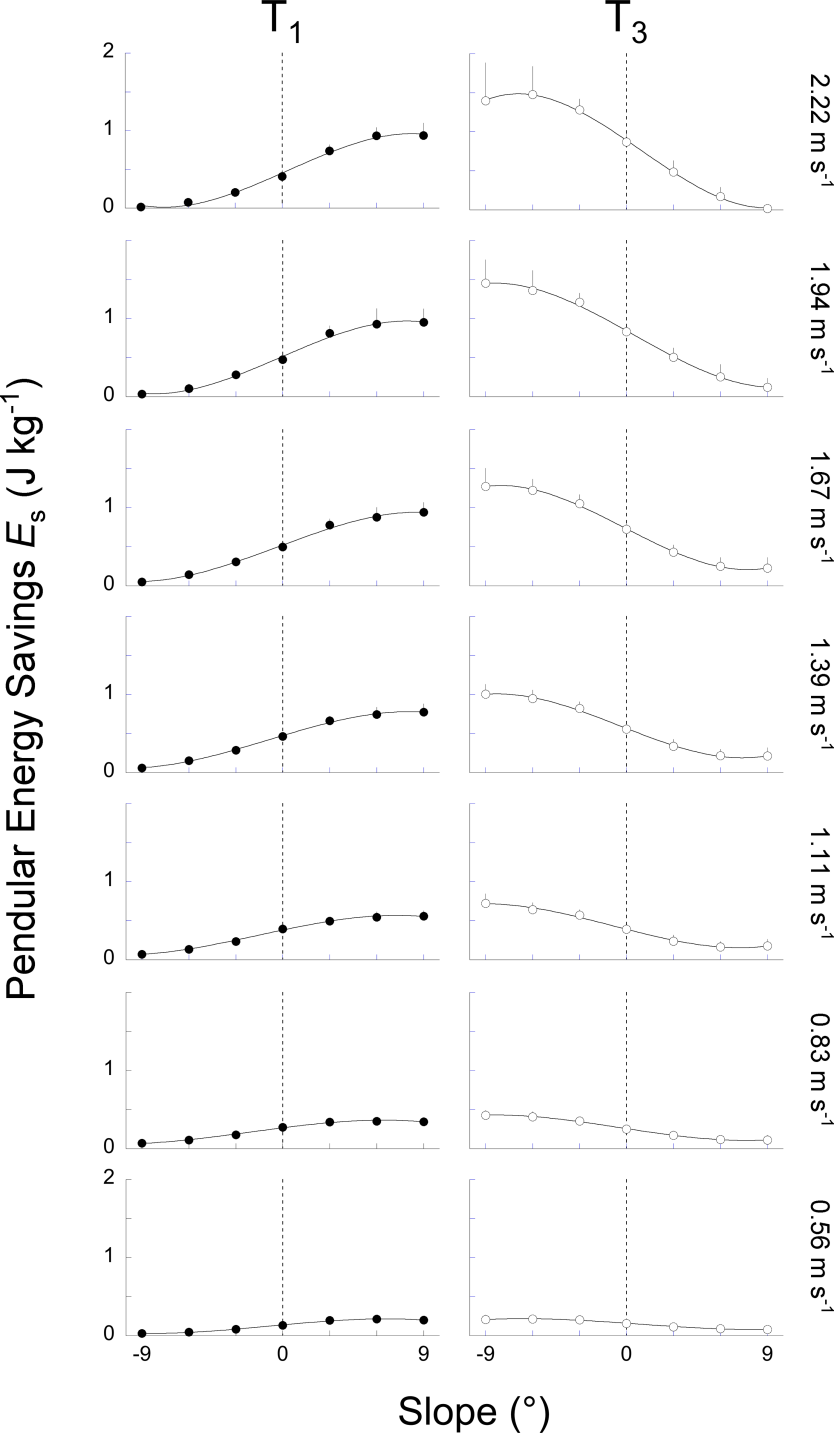
Pendular energy savings (*E*_s_) over the periods T1 and T3 as a function of slope at each speed. At each speed, the left panel (closed circles) presents *E*_s_ (see Methods) over the period T1 whereas the right panel (open circles) presents *E*_s_ over the period T3. Other indications as in Fig 4.

During T1, when walking uphill at low speeds, *R*_avg_ decreases with slope. Indeed, $W_{k}^{-}$ is close to zero at all slopes (Fig 7), thus there is only a small amount of kinetic energy change to be transformed into potential energy change and $W_{\mathrm{ext}}^{+}$ ≈ $W_{p}^{+}$. Since $W_{p}^{+}$ increases with slope, *R*_avg_ decreases (Eq 3). As speed increases, $W_{k}^{-}$ becomes greater; there is thus more and more kinetic energy change that can be transformed into potential energy and *R*_avg_ increases with slope. Since the duration of T1 tends to lengthen on steeper inclines, *E*_s_ during T1 increases with slope (Fig 9), even when *R*_avg_ decreases.

During T3, when walking uphill at speeds < 1.39 m s^-1^, *R*_avg_ is rather constant at all slopes (Fig 8) because $W_{p}^{-}$ is about equal to $W_{k}^{+}$ (Fig 7). Consequently, the *E*_ext_ curve is almost flat (Fig 1), *r*(*t*) reaches a peak close to 100% and *R*_avg_ is close to 50%. However, *E*_s_ is reduced as compared to level walking (Fig 9) because: (1) even if the *E*_k_ and *E*_p_ curves are out of phase, these curves change little (i.e. $W_{p}^{-}$ and $W_{k}^{+}$ are small), which limits the amount of energy that can be exchanged, and (2) the time during which this exchange can occur is reduced as compared to level walking. At speeds > 1.39 m s^-1^, *R*_avg_ decreases (Fig 8) because the *E*_p_ curve is increasing quasi monotonically (Fig 3). Consequently, $W_{p}^{-}$ tends to zero when slope increases (Fig 7) and the amount of potential energy that can be transformed into kinetic energy becomes smaller and smaller. Furthermore, the duration of T3 is shorter as slope increases. As a consequence, *E*_s_ becomes smaller on steeper slopes (Fig 9).

#### Downhill walking

When walking downhill, *R*_avg_ over T1 decreases from 0° to -9° and from 0.83 to 2.22 m s^-1^ (Fig 8). Furthermore, when negative slope increases, $W_{k}^{-}$ and $W_{p}^{+}$ tend to zero (Fig 7) and the duration of T1 decreases (Fig 6). Therefore, *E*_s_ during T1 diminishes with slope down to zero at -9° (Fig 9).

During T3, below 1 m s^-1^, *R*_avg_ decreases from 60% at 0° to <40% at -9° (Fig 8) because $W_{k}^{+}$ is much smaller than $W_{p}^{-}$ (Fig 7). Consequently, $W_{p}^{-}$ cannot be transformed in $W_{k}^{+}$ and *R*_avg_ is low. Above 1 m s^-1^, more and more potential energy can be transformed into kinetic energy (Fig 7) and *R*_avg_ increases mostly with speed (Fig 8): from ~65% at 1.11 m s^-1^ to ~80% at 2.22 m s^-1^. Furthermore, the duration of T3 becomes also longer when the negative slope increases (Fig 6). For these two reasons, *E*_s_ is greater on steeper than on shallow declines and at high than at low speeds (Fig 9).

### External work within the step

**Fig 10:**
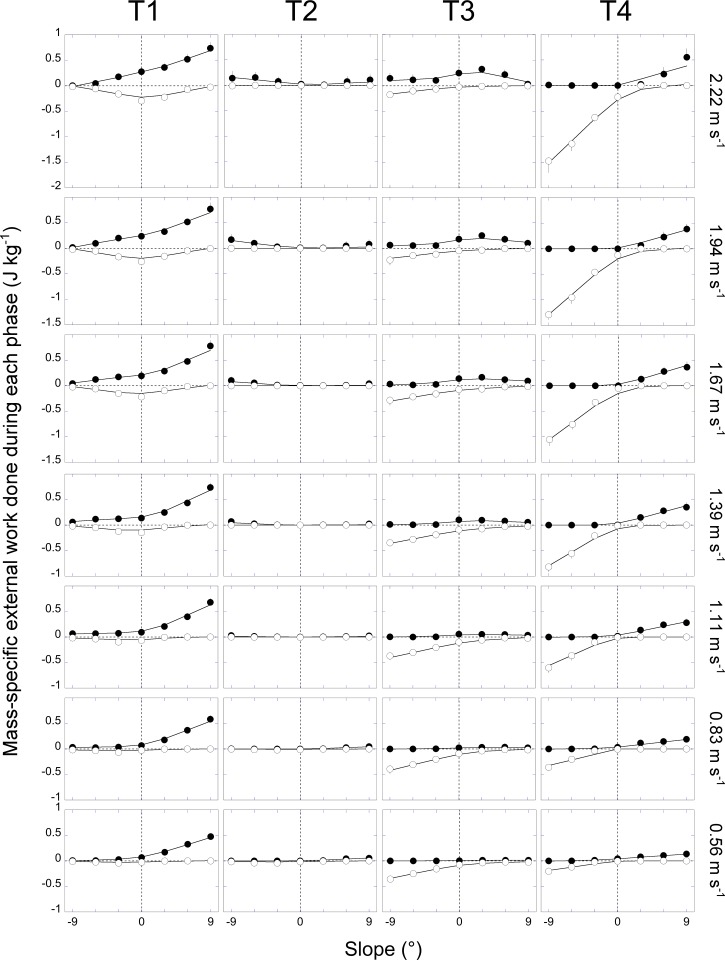
External work done during the four phases of the step as a function of slope at each speed. At each speed, the mass-normalized external work is presented during the four phases of the step as a function of the slope. In each panel, the closed and open circles correspond respectively to the positive and negative external work done during that phase. Other indications as in Fig 4.

Despite the fact that energy is saved during T1 and T3 through *E*_p_-*E*_k_ transduction, positive or negative work is still performed by biological tissues (i.e. muscles, tendons, ligaments, soft tissues) during each walking step (Fig 10). When walking uphill, the principal positive external work phase occurs during T4 and the following T1; this work increases with slope and speed of progression. On a shallow positive slope when walking faster than 1.5 m s^-1^, a small amount of negative external work is also done during T1. This negative work increases with speed and decreases with slope.

When walking downhill, the main negative external work phase occurs during T3 and the following T4. This negative work increases when negative slope increases. When speed increases, the negative work decreases during T3, while it increases during T4. On a shallow negative slope when walking faster than 1.2 m s^-1^, positive external work is also done during T1, this work increases with speed and decreases with slope.

Because T2 occurs around *E*_p,max_ and *E*_k,min_, the changes in amplitude of the *E*_p_ and *E*_k_ curves are small (Figs 1, 2 & 3). Furthermore, due to the short duration of T2 (Fig 6), the magnitude of COM work done during T2 is negligible (Fig 10).

## Discussion

In walking, the trajectory of the body tends to minimize the muscular work by limiting the absorption and production of *E*_p_ and *E*_k_ occurring each step [18]. The present study is intended to analyze the interaction between the forward and vertical motion of the COM by assessing the changes in pendulum-like exchange between *E*_k_ and *E*_p_ with the slope of the terrain and the speed of progression. Therefore, we employed estimates of the 'instantaneous' recovery and of the rate of pendular energy savings, within each step. During phases of perfect *E*_p_-*E*_k_ exchange (*i*.*e*. when *r(t)* = 100%), no muscle work is theoretically required to move the COM along its trajectory. However, a high *r(t)* does not imply a great amount of mechanical energy exchanged. Therefore, we introduce a new variable to measure the effectiveness of the *E*_p_-*E*_k_ transduction: the pendular energy savings *E*_s_. Furthermore, a high rate of energy savings does not imply *per se* a low amount of external work done (Figs 9 & 10). Therefore, we also evaluated the mechanical work required to sustain the motion of the COM throughout the step.

### Effect of slope and speed on the external work and recovery over the whole step

To allow comparison with earlier studies, the recovery over the whole step (*R*_step_) is computed here from the work associated with the horizontal (*W*_f_) and vertical (*W*_v_) movements of the COM. When walking on level ground, Cavagna et al. [1] showed that in walking the recovery of mechanical energy is maximal (i.e. about 65%) at 4–5 km h^-1^. Our data are consistent with these results but also with others (e.g. [1, 2, 6]): *R*_step_ reaches a maximum of ~65% around 1.39 m s^-1^. At that speed, the peak-to-peak amplitudes of *E*_p_ and of *E*_k_ are about the same (Fig 7) and the phase shift between *E*_p,min_ and *E*_k,max_ is about zero (Fig 6). At low speeds, the magnitude of *E*_p_ is greater than that of *E*_k_ and *E*_p,min_ precedes *E*_k,max_, yielding *R*_step_ values <60%. At high speeds, the amplitude of *E*_p_ is smaller than that of *E*_k_ and *E*_p,min_ follows *E*_k,max_, yielding *R*_step_ values <50%.

When walking on any slope at a steady speed, $W_{f}^{+}\approx W_{f}^{-}$ since the average acceleration of the COM in the fore-aft direction is nil over a complete step. However, at a given speed the magnitude of the *E*_kf_ curve varies across slopes. When walking downhill, $W_{f}^{+}$ and $W_{f}^{-}$ increases slightly (Fig 5). This observation is in agreement with previous studies showing that the peak of the horizontal component of the GRF is greater after foot contact when walking on negative slopes [19–21]. Conversely, $W_{f}^{+}$ and $W_{f}^{-}$ are smaller when walking uphill, especially at high speeds (Fig 4). These observations are in accordance with previous results reporting a smaller posterior shear GRF [20, 21]. The change in *W*_f_ with slope could be due to a modification of the distance between the COM and the front foot at foot contact [22].

When walking uphill, the increase in $W_{\mathrm{ext}}^{+}$ to ascend the slope is accompanied by a decrease in $W_{\mathrm{ext}}^{-}$ (Fig 4). Conversely, when walking downhill, the increase in $W_{\mathrm{ext}}^{-}$ is accompanied by a reduction of $W_{\mathrm{ext}}^{+}$. These results are in agreement with those of Minetti et al. [23]. The opposite change in the magnitude of $W_{\mathrm{ext}}^{+}$ and $W_{\mathrm{ext}}^{-}$ jeopardizes the *E*_k_-*E*_p_ transduction. When walking uphill, *R*_step_ is reduced, as compared to level. Our results corroborate those of Gomeñuka et al. [6] and of Gomeñuka et al. [7] obtained at speeds between 0.3 and 1.4 m s^–1^. When walking downhill, *R*_step_ is also reduced. Our results differ instead from those of Gottschall and Kram [5], who observed an increase in *R*_step_ after factoring out the changes in *E*_p_ due to slope. The reduction in *R*_step_ observed here fails to explain the reduction of the metabolic cost on gentle negative slope [24].

Furthermore, both on a positive and negative slope, *R*_step_ reaches a maximum at a higher speed than on level walking (Figs 4 & 5). One reason could be the following: on a slope, the changes in the *E*_p_ curve increase mainly with slope while the *E*_k_ curve increases with speed. Consequently, when slope increases, the possibility of maximal exchange between *E*_p_ and *E*_k_ occurs at a higher speed.

When walking on the level, the speed at which the metabolic costs is minimal (i.e. the optimal metabolic speed) corresponds approximately to the speed at which *R*_step_ is maximal [6] and *W*_ext_ is minimal [1, 17]. Therefore, the optimal metabolic speed is often explained by a minimal external work, which in turn is linked to an optimal recovery. When walking on a slope, the optimal metabolic speed decreases from -9° to +9° [6, 25]. On the contrary, both in uphill and downhill walking, *R*_step_ reaches a maximum at higher speeds than on the level (Figs 4 & 5) and *W*_ext_ does not present anymore a minimum. Therefore, the relation between optimal metabolic speed, minimum external work and maximum recovery seems to cease, undermining the usefulness of the pendular transduction analysis to predict metabolic energy expenditure.

While *R*_step_ characterizes energy exchange in walking, it cannot be used to identify functionally distinct gaits; for example, many different types of animal gait exhibit the same recovery [26, 27]. In humans, gaits with orthopedic pathologies [28] or hemi-paretic walking [29] did not highlight the expected reduction in *R*_step_. This lack in sensitivity could be due to the fact that *R*_step_ is measured over the whole walking step and does not give information about the time-course of the energy transduction within the step.

### The compass gait model during walking on a slope

At first approximation, the trajectory of the COM during walking on the level can be described by a ‘compass gait’ model (e.g. [30–33]). In this case, the arc of the ‘compass gait’ model is more or less symmetric with respect to the vertical (see schemas in Figs 1, 2 & 3). Here we adapt this ‘compass gait’ model to illustrate how and why the *E*_k_-*E*_p_ transduction is reduced when walking on a slope at different speeds.

On a positive slope, the changes in the pendulum-like energy exchange can be illustrated by tilting the arc of the compass backwards (Figs 1, 2 & 3). As a consequence, the lower-limb reaches its vertical position later during the contact phase. As speed and slope increase the duration of T1 becomes longer and T3 becomes shorter than at 0° slope (Fig 6). Furthermore, when the arc is inclined to the back, its end portion is oriented more horizontally while its first portion is more vertical. Consequently, when the positive slope becomes steeper, the vertical movement of the COM increases during T1 and decreases during T3 (Fig 7). This means that most of the energy recovery during the step occurs during the phase T1, where $W_{k}^{-}$ is transformed in $W_{p}^{+}$ to raise the *COM*. As speed and slope increase, both $W_{k}^{-}$ and $W_{p}^{+}$ increase, which enhances the possibility of energy transformation. Consequently, *E*_s_ increases with speed and slope during T1; however, the recovery is not faultless and positive work is still performed to elevate the COM (Fig 10). During T3, as speed and slope increases, *E*_s_ trends toward zero because $W_{p}^{-}$ and $W_{k}^{+}$ become smaller and smaller and the duration of this phase shortens, due to the tilt of the compass.

On a negative slope, the changes in the pendulum-like energy exchange can be illustrated by tilting the arc of the compass forwards (Figs 1, 2 & 3). Consequently, the lower-limb reaches its vertical position earlier during the contact phase, as also shown by Gottschall and Kram [5], so that the duration of T1 becomes shorter and the duration of T3 longer. Furthermore, when the arc is declined to the front, its first portion is oriented more horizontally while its last portion is more vertical. This means that most of the energy transformation is done during T3, when the COM falls and accelerates forward. Since $W_{k}^{+}$ increases with speed and $W_{p}^{-}$ with slope (Fig 7), *E*_s_ during T3 is greater when walking on a steep terrain at high velocity (Fig 9). During T1, *E*_s_ is limited and trends toward zero as speed and slope increase, since the duration of this phase as well as the magnitude of $W_{p}^{+}$ and of $W_{k}^{-}$ diminish, due to the tilt of the compass.

Our results suggest that at each slope the arc of the compass and consequently the position of the COM relative to the foot at mid-stance are tilted relative to level walking (Figs 1, 2, 3 & 6). These observations are in accordance with the gait kinematics data of Lay et al. [20] and McIntosh et al. [21].

### Effect of slope and speed on the external work during the phases of the step

During T2 and T4, the *E*_k_ and *E*_p_ curves are in phase and thus no *E*_k_-*E*_p_ exchange is possible. The phase T2 occurs during single-contact around *E*_p,max_ and *E*_k,min_. At all speed and slopes, the duration of T2 is short or even nil sometimes [8]. Consequently, the external work is close or equal to zero. The phase T4 occurs around *E*_p,min_ and *E*_k,max_. When walking on a flat terrain, T4 happens during the double contact phase, as the leading leg performs negative work while the trailing leg generates positive work [34–37]. At low speeds, net positive external work is done during T4 to accelerate and redirect the COM upward [38], whereas at high speeds, net negative work is done to decelerate and lower the COM. Although brief, T4 is crucial because the action of the muscle-tendon units during the double-support phase establishes the initial conditions for the subsequent single support phase [39]. Using a simple relationship between muscular work and energy consumption, Yeom and Park [40], Kuo et al. [41] and Ruina et al. [42] have suggested that the most economical way of walking on the level would be to compensate the collision loss after foot contact in the leading limb by push off work of the trailing limb around the double support phase and to perform limited (or zero) additional work during the single support phase.

In uphill walking, the duration of T4 is extended because *E*_p,min_ appears increasingly earlier in the step as slope increases (Fig 6). Functionally, a longer T4 indicates that the COM is still rising when its forward velocity starts to increase (Figs 1, 2 & 3). Walking uphill on steeper slopes involves a greater $W_{\mathrm{ext}}^{+}$ during double support and this positive work is done both by the leading and the trailing legs [43, 44]. Our results show that, a significant part of the positive external work performed during the step is generated during T4 and T1 and that this work increases when speed increases (Fig 10). These results are in accordance with those of Gottschall and Kram [45]. According to Oh et al. [46], from a purely mechanical perspective, the external work would be minimal if the positive work done during the double support phase was equal to the potential energy necessary to overcome the slope and was followed by a single support phase during which the COM vaults passively over a stiff limb. However, generating a large magnitude of work over a short time duration may be metabolically expensive for muscles [45] and most likely exceed a maximum suitable push-off limit [46]. Instead, to prevent pitching backward when moving on an incline, the hip and knee flexion increases at foot contact [47, 48] and mechanical work is performed to raise the COM of the body during T1 when extending the knee and hip [21]. During T1, up to 60% of the increase in *E*_p_ comes from the decrease in *E*_k_. Additional positive muscle work is also performed during this period, incurring a metabolic energy cost [49].

In downhill walking, the duration of T4 is extended because *E*_p,min_ appears later and later in the step as slope increases (Fig 6). Functionally, a longer T4 indicates that the COM is still lowering when its forward velocity starts to decrease. Walking downhill on steeper slopes and at higher speeds involves greater negative external work during double support (Fig 10) and this work is mainly done by the leading leg but also by the trailing leg [43, 44]. Similar to uphill walking, the external work performed during downhill walking is predicted to be minimal if the negative work done during the double contact was equal to the decrease in potential energy due to the slope and if no additional work was done during the single support phase [46]. Our results show that, at low walking speed, *R*_avg_ is small during T3 suggesting that negative muscle-tendon work is performed to control the velocity of the COM during the descent (Fig 10) and to restrain the body from falling [50]. As speed increases, *R*_avg_ during T3 increases up to ~80% so that most of the kinetic energy required to move the COM forward comes from transduction of potential energy [31, 41, 51]. Consequently, the negative work performed during T3 tends to zero and the major part of the energy dissipation occurs during T4. These results are in agreement with those of Gottschall and Kram [45] and Lay et al. [52] and suggest that the major contribution of biological tissues to absorb energy occurs around the step-to-step transition.

### Limitations of the study

The pendular transduction analysis performed in this study could not shed any light on the relation between mechanical external work and metabolic energy expenditure (for a discussion of this issue, see [5, 6, 23, 25]). However, external work does not represent the total work done, which also includes (among others) the internal work done to move limbs relative to the COM [16, 23, 53–55] and the work done by the trailing limb against the leading limb during double support [35, 56]. Unfortunately, the method used here does not allow measuring individual limbs *GRF*. Therefore, we cannot take into account the simultaneous mechanical work performed by each limb during the step-to-step transition [35]. Furthermore, metabolic cost is a function of work but also of other factors like force and rate of force production [57], whether work is from muscle, tendon or soft tissue [58, 59], which muscles are active and what part of the force-length and force-velocity curves a muscle is on.

## Conclusion and perspectives

The present study characterizes the within-step recovery *r*(*t*) and a new outcome metric, the theoretical pendular energy savings *E*_s_, to analyze human walking biomechanics. These continuous within-step estimates offer the possibility to determine the phase of the step during which motion of the COM exhibits pendulum-like energy transduction. Our study shows that during uphill walking, the pendular energy savings are larger during the first part of stance phase (T1), when kinetic energy is transformed into potential energy. On the contrary, during downhill walking, the pendular energy savings are larger during the second part of stance (T3), when potential energy is transformed into kinetic energy. Both in uphill and downhill walking, the main phase of external work production/absorption occurs around the double support phase. The changes observed in the pendulum-like energy exchange with slope and speed can be illustrated by tilting the 'classical compass model' backwards (uphill) or forwards (downhill). Future research should link this tilting behavior with the changes in the kinematics of the limb-segments and should correlate the modifications of the mechanical demand with the changes in the muscular activity.

## Supporting information

S1 Dataset All_Data_Dewolf. Data of all subjects in each condition.
(XLSX)

## Abbreviation

*a*_f_: horizontal component of the acceleration of the COM in the fore-aft direction
*a*_l_: lateral component of the acceleration of the COM
*a*_v_: vertical component of the acceleration of the COM
COM: center of mass of the whole body
External work: work necessary to move the COM relative to the surroundings
*E*_ext_: mechanical energy of the COM
*E*_kf_, *E*_kl_, *E*_kv_: kinetic energy due to the fore-aft, lateral and vertical movements of the *COM*
*E*_k_, *E*_p_: kinetic and potential energy of the COM
*E*_k,min_, *E*_k,max_: local minimum and maximum of the *E*_k_-time curve
*E*_p,min_, *E*_p,max_: local minimum and maximum of the *E*_p_-time curve
*E*_s_: pendular energy savings, i.e. amount of energy saved over a given period of time through the *E*_k_-*E*_p_ transduction
$\dot{e}(t)$: rate of pendular energy savings: amount of energy economized through the transduction between *E*_p_ and *E*_k_ divided by the time between two samples
*F*_f_: horizontal component of the GRF in the fore-aft direction
*F*_l_: component of the GRF parallel to the short axis of the tread-surface
*F*_n_: component of the GRF perpendicular to the tread-surface
*F*_p_: component of the GRF parallel to the long axis of the tread-surface
*F*_v_: vertical component of the GRF
g: acceleration of gravity
GRF: ground reaction force
m: body mass
*R*_avg_: percentage of energy recovered during a phase of the step through the transduction between *E*_p_ and *E*_k_
*R*_step_: percentage of energy recovered during the whole step through the transduction between *E*_p+_*E*_kv_ and *E*_kf_
*r*(*t*): instantaneous recovery: percentage of energy recovered between two samples through the transduction between *E*_p_ and *E*_k_
t: time
T1: period of the step during which *E*_p_ increases while *E*_k_ decreases, i.e. period more or less during the first part of the contact phase when the COM increases its height
T2: period of the step during which *E*_p_ and *E*_k_ are in phase, period between T1 and T3, i.e. more or less in the middle of the contact phase
T3: period of the step during which *E*_p_ decreases while *E*_k_ increases, i.e. period more or less during the second part of the contact phase when the COM loses height
T4: period of the step during which *E*_p_ and *E*_k_ are in phase, period between T3 and T1, i.e. more or less at the very end of the contact phase
θ: slope of the treadmill, i.e. angle between horizontal and the tread-surface
*v*_belt_: instantaneous velocity of the belt of the treadmill
*v*_f_: horizontal component of the velocity of the COM in the fore-aft direction
*v*_l_: lateral component of the velocity of the COM
*v*_v_: vertical component of the velocity of the COM
$W_{\mathrm{ext}}^{+},\;W_{\mathrm{ext}}^{-}$: positive and negative work due to the time-changes in *E*_com_
$W_{f}^{+},\;W_{f}^{-}$: positive and negative work due to the time-changes in *E*_kf_
$W_{k}^{+},\;W_{k}^{-}$: positive and negative work due to the time-changes in *E*_k_
$W_{l}^{+},\;W_{l}^{-}$: positive and negative work due to the time-changes in *E*_kl_
*W*_min_: minimal work done per unit distance to overcome the slope
$W_{p}^{+},\;W_{p}^{-}$: positive and negative work due to the time-changes in *E*_p_
$W_{v}^{+},\;W_{v}^{-}$: positive and negative work due to the time-changes in *E*_p_*+E*_kv_
